# Supplementary material for: Host interactions of novel Crassvirales species belonging to multiple families infecting bacterial host, Bacteroides cellulosilyticus WH2
Source: Microb Genom. 2023 Sep 4;9(9):001100. doi: 10.1099/mgen.0.001100 (PMC10569736; doi:10.1099/mgen.0.001100)
Supplement: Supplementary material 5 [file mgen-9-1100-s006.pdf]

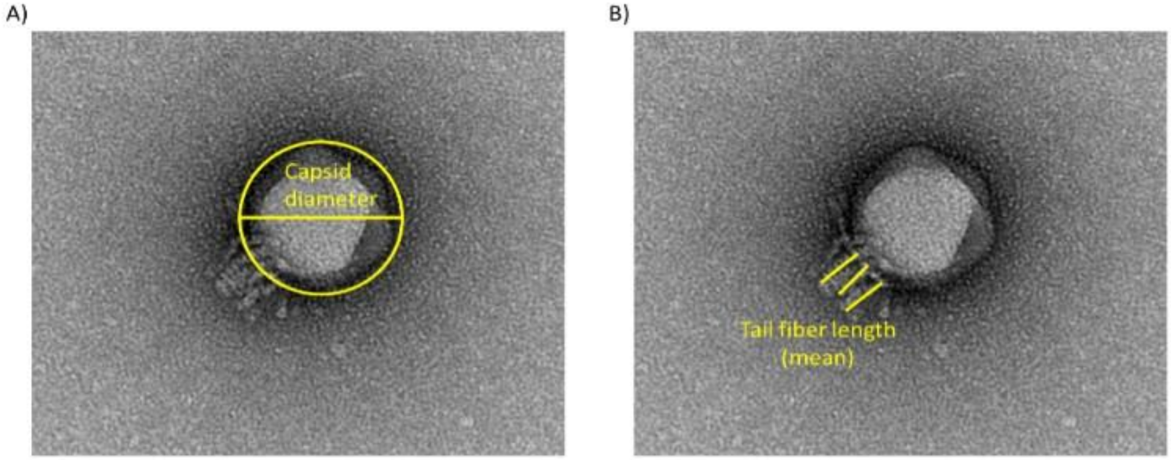

Fig. S1. TEM phage measurements were taken for A) Capsid diameter, by drawing a circle around the polygon with the edges within the circle. The diameter of this circle was measured and represented as the capsid diameter. B) For tail length, a line was drawn from the base of the capsid to the visible edge of the tail fibers. This was repeated over five phages of the same sample and an average with standard deviation was calculated across all of them.

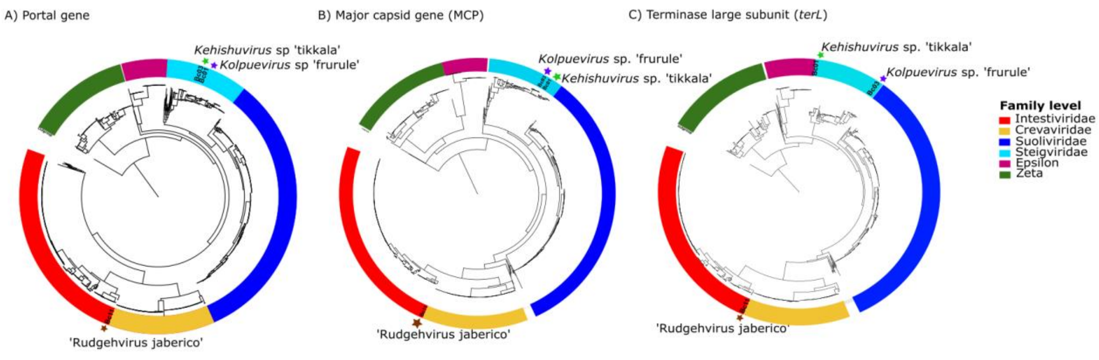

Fig. S2. Showing the taxa classification of the three novel species remains consistent across the three conserved proteins A) portal gene, B) Major capsid protein (MCP), and C) terminase large subunit (terL). The outgroup across all three trees set to *Cellulophaga phage* phi13:2. The placement of the three novel species are highlighted on the tree, Bc01 belonging to *Kehishuvirus* genera (light green), Bc03 belonging to *Kolpuevirus* genera (purple), and Bc11 belonging to a novel genus named ‘Rudgehvirus’ (brown). TEM phage measurements were taken for A) Capsid diameter, by drawing a circle around the polygon with the edges within the circle. The diameter of this circle was measured and represented as the capsid diameter. B) For tail length, a line was drawn from the base of the capsid to the visible edge of the tail fibers. This was repeated over five phages of the same sample and an average with standard deviation was calculated across all of them.

```
Reference sequence (1): WEU69859.1-Bc11
Identities normalised by aligned length.
Colored by: consensus/70%

                          cov     pid     1 [        .         .         .         .         :         .         .         .         .         1
1 WEU69859.1-Bc11     100.0%  100.0%        ---METNNRK------QLVTQDNGNIEKIFPKNYMSNIVDEETGESLRI----------------FLLKYNHIDLGYRSSKSSARLAVPIIMRRKGLYIT
2 WEU69744.1-Bc01      60.8%   38.5%        MYFTQEDYRKIEKWLLANSKKDTQFVEAATPLQGNETIVLVQGGKNVNTTVSDIVSQFFALGVSDFINVTDKYGISYNTLDEAIRV-IPWRSRKVGQVIT
3 WEY17522.1-Bc03      60.8%   38.5%        MYFTQEDYRKIEAYLKSKAARDTSFDSATTPLQGNETIVLVQGGKNVNTTVSDIVSQFFALGVSDFINVTDKYGISYNTLDEAIRV-IPWRSRKVGQVIT

                          cov     pid   121          .         .         :         .         .         .         .         2         .         .
1 WEU69859.1-Bc11     100.0%  100.0%        SDTNWVKDELWVKDVNELNVYDVKISDGSITLDKLSEEVMQLISSKGDITINNFPDNEDLELYTIYQDSNNKIDAIRFKDRDNS----NGMAYKYLRKTK
2 WEU69744.1-Bc01      60.8%   38.5%        --LTWNNTTLWV--------------------DLLESRIVNSI----------LPDQEDLTMTEPDADGNS---YMHFKDKDYSIDDFSGLGGVYLRKNL
3 WEY17522.1-Bc03      60.8%   38.5%        --LTWNNTTLWV--------------------DLLESRIVNSI----------LPDQEDLTMTEPDADGNS---YMHFKDKDYSIDDFSGLGRVYLRKNL

                          cov     pid   241          :         .         .         .         .         3         .         .         .         .
1 WEU69859.1-Bc11     100.0%  100.0%        DFNQANTIYEIRYDFDLNGSTISIPDNCELKFIGGSLKNGTINFNNTLLNGNIKIKTNISGNIRNNEVNVLWFNVTGDGNADDRNAIQEVIDIVSESTTI
2 WEU69744.1-Bc01      60.8%   38.5%        MVGKENVIYHIQYDYNLNGQTIIIPEGCVLLFEGGSISNGTINFSNTFLTGDIKIKTDLLGTIKNNSLNVLWFNVTGDGNTDDRKAIQDIFNIAASNTTV
3 WEY17522.1-Bc03      60.8%   38.5%        MVGKENVIYHIQYDYNLNGQTIIIPEGCVLLFEGGSISNGTINFSNTFLTGDIKIKTNLLGTVKNNSLNVLWFNVTGNGNTDDRKAIQDVFNIASSNTTV

                          cov     pid   361          .         .         .         4         .         .         .         .         :         .
1 WEU69859.1-Bc11     100.0%  100.0%        TIEQRANAVYSGSLYPLTINKNNITIVIRGIIKSTNILGNMFEVSGNNIVIIGEDGKLQGIGGTDNEGFLDTNTSDTTLQWHPCLIKINGDNCRINNLSF
2 WEU69744.1-Bc01      60.8%   38.5%        IIDQRANAVANNTLYPITVSNDNVDIVIKGTIKSTTILGNMFELSGNNISLLGEGGKLQGPGGADDVGFLDTNTADTALQWHPCLLKVDGNNCRVSNLSF
3 WEY17522.1-Bc03      60.8%   38.5%        IIDQRANAVANNTLYPIIVSNDNVDIVIKGIIKSTTILGNMFELSGNNIVLLGEGGKLQGPGGADDVGFLDTNTADTALQWHPCLLKVDGNNCRVSNLSF

                          cov     pid   481          .         5         .         .         .         .         :         .         .         .
1 WEU69859.1-Bc11     100.0%  100.0%        LFIGGRIEHSTTGGTVLMGIRTNTGADSVIIKNNRFIANPKGGKCYSCIFPSGDKFIISNNICEKYHEHAVYAYGNYGSIIDNKFTDVDCIASDIQYFAT
2 WEU69744.1-Bc01      60.8%   38.5%        LFIGGRVNHSTTGGTVLMGIRTNVGADSAIIKNNRFIANSIGGKCYSCVFPSGNNFIISDNICEKYHEHAVYAYGDYGSIFNNKFTDVDCIASDIQYFAT
3 WEY17522.1-Bc03      60.8%   38.5%        LFIGGRVNHSTTGGTVLMGIRTNVGADSAIIKNNRFIANSIGGKCYSCVFPSGNNFIISDNICEKYHEHAVYAYGDYGSIFNNKFTDVDCIASDIQYFAT

                          cov     pid   601          .         .         .         .         :         .         .         .         .         7
1 WEU69859.1-Bc11     100.0%  100.0%        GKGTIVEGNTVTNCRGGIIVRPYHTVDVDKLPSGLKLLNNFIELTVGTTGSCLSFYVLGTYDISDILISGNTFANGYYTQTSQVAASLLIGAGSSENNGI
2 WEU69744.1-Bc01      60.8%   38.5%        GKGTIVEGNIATGCRGGIIVRPYHTVDVTKLPSGLKIVNNFIELSIGTTGSCLSFYVVGTYDISDILIAGNTFSNGYYTQTSQVAASLLIGAGSSGNNGI
3 WEY17522.1-Bc03      60.8%   38.5%        GKGTIVEGNIATGCRGGIIVRPYHTVDVTKLPSGLKIVNNFIELSIGTTGSCLSFYVVGTYDISDILIAGNTFSNGYYTQTSQVAASLLIGAGSSGNNGI

                          cov     pid   721          .         .         :         .         .         .         .         8         .         .
1 WEU69859.1-Bc11     100.0%  100.0%        KGIHNSIFKNNKFISESLNDVASAFSFAINANYVTNTIFEGNEFIDVKDTPTLARAIQLNTGTTNLSVIDNNIINYTSNIYLDYDTYTKLIYKRNNRRNY
2 WEU69744.1-Bc01      60.8%   38.5%        TGIHNSIFKNNKFISESLDGVTSAFSSAINANYVTDTIFEGNEFIDIKSTPTMARAIQLNTGTTNLTVADNNIINYTSNIYLDYDTDAKLIYKRNNRLNY
3 WEY17522.1-Bc03      60.8%   38.5%        TGIHNSIFKNNKFISESLDGVTSAFSSAINANYVTDTIFEGNEFIDIKSTPTMARAIQLNTGTTNLTVADNNIINYTSNIYLDYDTDAKLIYKRNNRLNY

                          cov     pid   841          :         .         .         .         .         9         .         .         .         .
1 WEU69859.1-Bc11     100.0%  100.0%        EYYDKDNQVPMWWSGVRWKDAEGCYIVLSKSDINTPNKIYKIRFNIDLGGTTLTLPANCTLDFQGGSFSNGTIVGSNTRFSGNIKLDTTLTFSGTYLLDT
2 WEU69744.1-Bc01      60.8%   38.5%        NYFDTDLMKPMWWSGVAWRDADG-----------------------------------------------------------------------------
3 WEY17522.1-Bc03      60.8%   38.5%        NYFDTDLMKPMWWSGVAWRDADG-----------------------------------------------------------------------------

                          cov     pid   961          .         .         .         0         .         .         .         .         :         .
1 WEU69859.1-Bc11     100.0%  100.0%        ALDFAKLIKASVTFGNKLTSGYIIKTTLNCDVPIIGNNARLSSTTVGVLNCKNECSIENLYIDFYPYQGASDVTDNKVIAINIGGETSTYNHYINNVTID
2 WEU69744.1-Bc01      60.8%   38.5%        ----------------------------------------------------------------------------------------------------
3 WEY17522.1-Bc03      60.8%   38.5%        ----------------------------------------------------------------------------------------------------

                          cov     pid  1081          .         1         .         .         .         .         :         .         .         .
1 WEU69859.1-Bc11     100.0%  100.0%        IIRTRIGIQAYGKCVNNSIVNANIDCKGTNSICIAFNDEVDHAASTYLSEGWTISNSILTSAQYGIFGVNMSNTFISNCIIDLCSKNAIKFSLNSLNIRI
2 WEU69744.1-Bc01      60.8%   38.5%        ----------------------------------------------------------------------------------------------------
3 WEY17522.1-Bc03      60.8%   38.5%        ----------------------------------------------------------------------------------------------------

                          cov     pid  1201          .         .         .         .         :         .         .         .         .         3
1 WEU69859.1-Bc11     100.0%  100.0%        NVLNNEDAGIIISNNTIKSYYSDSGSCNGINIGEGTYESIYILGNTISNLGGIGIVSLNGEITHINVSDNRFKINVGGYFYVLKATNKYIANNNAIDNYN
2 WEU69744.1-Bc01      60.8%   38.5%        ----------------------------------------------------------------------------------------------------
3 WEY17522.1-Bc03      60.8%   38.5%        ----------------------------------------------------------------------------------------------------

                          cov     pid  1321          .         .         :         .         .         .         .         4         .       ] 1
1 WEU69859.1-Bc11     100.0%  100.0%        NTKTWNAGDICLNSNASVGSIIGWFCATSGSPGTWFPIGTIEQYAGLKKQGTTAERPAMNQNYIGATYYDTTLKKVILWNGTTWTNVDGTALT-----
2 WEU69744.1-Bc01      60.8%   38.5%        -----------------------------------YPINT--------KVGTTAQRPSLASTFVGFEYFDTTLNAPIMWDGTKWINEDGTLTSKVVIV
3 WEY17522.1-Bc03      60.8%   38.5%        -----------------------------------YPINT--------KVGTTAQRPSLASTFVGFEYFDTTLNAPIMWDGTKWINEDGTLTSKVVIV
```

MView 1.63, Copyright © 1997-2018 Nigel P. Brown

Fig. S3. Multiple sequence alignment of shared structural protein 1 from Fig 2C including ‘K. tikkala’ strain Bc01 protein, WEU69744.1, ‘K. frurule’ strain Bc03 protein, WEY17522.1, and ‘R. jaberico’ strain Bc11 WEU69859.1 (reference sequence), showcasing the sequence identity with amino acids that were not shared in grey, and the rest in different color, based on the amino acid group.

```
Reference sequence (1): WEU69857.1-Bc11
Identities normalised by aligned length.
Colored by: identity

                         cov    pid    1 [         .         .         .         .         :         .         .         .         .         1         .
1 WEU69857.1-Bc11 100.0% 100.0%        MKTQKQDLGKVSLTCNGTWDAGKQYYRLCIVNDGNFASYISKK--DVPIGTVLSDERFWQPIANLRDDIKIDYETFKKEWLELLASIQIKLRSARVVVANEEARNNLTWLEV
2 WEU69745.1-Bc01  91.9%  44.1%        -MMENQQLIKKDRSANS-------YSKIFP---WTFTDLVLDRVTKESLDNILVRNNF----------IALPYVGSKAA-----TRLQVPMKNRRRGI----------WLSY
3 WEY17523.1-Bc03  90.7%  47.1%        -MMENQQLIKKDRSANS-------YSKIFP---WTFTDLVLDRVTKESLDNILVRNNF----------IALPYVGSKAA-----TRLQVPMKNRRRGI----------WLSY

                         cov    pid  121           .         .         :         .         .         .         .         2         .         .         .
1 WEU69857.1-Bc11 100.0% 100.0%        LDTQLTYILDSIVPVINLKSWHLVVGSLLDSEAKFQLDGTYELTAERAIADRWGYIIDEVYVSKKEVKNLVLSIVNDKLENMSITIPPNSITPEDLSQAVLDLIGSGGNVVN
2 WEU69745.1-Bc01  91.9%  44.1%        GTLTVEYYNDN---NLDDNHWQ--------------DSSY-----------W-------------------------LPYNTAEFQPASVGLSALAQEVFDWINSQITTAV
3 WEY17523.1-Bc03  90.7%  47.1%        GTLTVEYYNDN---NLDDNHWQ--------------DSSY-----------W-------------------------LPYNTAEFQPASVGLSALAQEVFDWINSQITTAV

                         cov    pid  241           :         .         .         .         .         3         .         .         .         .         :
1 WEU69857.1-Bc11 100.0% 100.0%        SESLSNGTSVLKFKDRDYVEGTFNGLGEVILRKNQVGIINLLEQANINKPNTVYVVRYDFCLGGATITLPKNSTLKFEGGSIDNGTIVGNNSCIISDIDKTILGKDLVIEGT
2 WEU69745.1-Bc01  91.9%  44.1%        ----KNSSGQIEEANRAYDTSTFSGLGYRILRKNIQSNKNILTQSMINMPNNVYKIRYDFDLNGATINLPANSVLQFVGGSIKNGTLNGNNTVIEADSNAVIF-DSVVIEGT
3 WEY17523.1-Bc03  90.7%  47.1%        ----KNSSGQIEEANRAYDTSTFSGLGYRILRKNIQSNKNILTQSMINMPNNVYKIRYDFDLNGATINLPANSVLQFVGGSIKNGTLNGNNTVIKADSNAVIF-DSVVIEGT

                         cov    pid  361           .         .         .         4         .         .         .         .         :         .         .
1 WEU69857.1-Bc11 100.0% 100.0%        KWFAFDSSADFLSNQIITNILALSNDNVYNTIHFDADRTYYFENTYKGKTNLGDDVRPNYWLLNTPDYDFLRIFTGFTSNTHLIVNNTIQMLPTNQGAYFIFHIENKENITI
2 WEU69745.1-Bc01  91.9%  44.1%        SWFAFNTSPSYISNQIITNILALSNDDVYNTIHFDADRTYYFELPYKGRANLGDDVRPDYWKLNTEEYSFLRIFTNFTSNTHLIVNNTIQMLPTNQGAYFIFHIEGKSNIEI
3 WEY17523.1-Bc03  90.7%  47.1%        SWFAFNTSPSYISNQIITNILALSNDDVYNTIHFDADRTYYFELPYKGRANFGDDVRPNYWKLNTEEYAFLRIFTGVTSNTHLIFNNTLQMIPTNQGAYFIFHIESKENIQI

                         cov    pid  481           .         5         .         .         .         .         :         .         .         .         .
1 WEU69857.1-Bc11 100.0% 100.0%        DAKDHLYTDPFAGTNYYGEWGHVLNFRSCNNVVVRDITIGYAFGDGIALGNAAYNNSGVKEAGLATKNVTIDAVKVLYARRNGISLGGNNYTITNVYFEGNGSDTIKGTAPM
2 WEU69745.1-Bc01  91.9%  44.1%        DAKDHLYTDPFAGANYYGEWGHIFNFRSCDNIVIRDITVGYAFGDALAFSNIAYNNNGTKAAGPATKNVLIDGVKVLYARRNGIALGGNNYTITNVYFEGCGSDEIKGTAPM
3 WEY17523.1-Bc03  90.7%  47.1%        DAKDHLYTDPFAGNNYYGEWGHVLNFRSCNDVIIRDITVGYAFGDGISLGNASYNNNGTKAAGLATKNVTVDGVKVLYARRNGISLGGNNYSITNVYFEGNGSDTIRGTAPM

                         cov    pid  601           .         .         .         .         :         .         .         .         .         7         .
1 WEU69857.1-Bc11 100.0% 100.0%        YVDIEPSGLCTNVSMNNCKFKDNKYDVSSTIRDDLYEVPRGELVNISDCNFTSPLRLNRTNGLTFSNCHIVGITNVDNSIAAWYVSKDLVFNSCIFDELNPYLAISAEEQNK
2 WEU69745.1-Bc01  91.9%  44.1%        YTEVEPSGVCSNVSMSSCKFKNNKYDVSSTIRDDLGPVPRGQLVTISDCNFTSPLRLNRTNGLTFNNCHIVGISNHDNSISPWFASKDLVFNSCVFDELNPYLAISAEEQNK
3 WEY17523.1-Bc03  90.7%  47.1%        YVDIEPSGLCKNVSMSNCKFKDNKYDVSSTIRDDLYEVPRGELVNISDCNFTSPLRLNRTNGLTFSNCHIVGITNVDNSVAAWYSSKDLIFSNCIFDELNPYLTISAEEQDK

                         cov    pid  721           .         .         :         .         .         .         .         8         .         .         .
1 WEU69857.1-Bc11 100.0% 100.0%        EDIRYSTTFQHNLPVGKALKFTIPKPLVGEVELTAFCSNPNYSAIQMPINTTIYTFGPSQRLTGIRDIKIKASQDSTPRYSMYKNTPVFSYINYTEDSNNFIIYFAIGGDLI
2 WEU69745.1-Bc01  91.9%  44.1%        EDIKYSTTFQQSLAAGRALKFTIPKPLVGLVEFTAFCSNPNYSAVQMPINITEYSFGNSQRLTGIRDIKVKAAQDNTLRYAIYRNVPVFSYINYSEDADNFNIYFAIGGDLI
3 WEY17523.1-Bc03  90.7%  47.1%        EDIRYTTTFQQNMAVGRALKFTIPKPLVGEVEFTAFCSNSNYSLIQMPINTTVYTFGPSPRLTGIRDVKIKASQDSTPRYFLYKNTPVFSYINYTEDTNNFIIYFAIGGSLI

                         cov    pid  841           :         .         .         .         .         9         .         .         .         .         :
1 WEU69857.1-Bc11 100.0% 100.0%        SVNIFLTSKTKFIIVEAPVSGRPDYAGMYGGKWSELSAIIKESVEISSIPSTVTFPSKEMYSGNMLADLPTSLTADKVGFSQFVLDSTYKRPVFWDSYSNVFRTADGNKALE
2 WEU69745.1-Bc01  91.9%  44.1%        SVNIFLTSKTKFIVVEDPVSENPGVAGMYGGKWSTLSAITKSVIDVTAIPSSVTFPSKEMFSANMLADLPTSLTADKVGFSQYVLDNSYKCPVFWDSFSSVFRTADGNRALV
3 WEY17523.1-Bc03  90.7%  47.1%        SVNIFLTSKTKFITIEAPVSGRPDYAGMYGGKWSQLSSIVKESIKVSDIPETVTFPSKELYEANLLADMPDNLTSDKVGKSVFVLDDTYRRPAFWDSYSNTFRTADGNRLYE

                         cov    pid  961           .         .         .         0         .         .         .         .         :         .         .
1 WEU69857.1-Bc11 100.0% 100.0%        LDVLTAKLT-VDDRGYVVYYTVTTSYLTWRGTDWTNEDGSLFSKVKYIKQTNTIDILNIMFSYSGVIYKICADIDLGGGTLTIATGSTLDFQGGSFNNGTIIGQNTKIESGL
2 WEU69745.1-Bc01  91.9%  44.1%        LNTLTDQMT-IDDRGYVVYTTVNNSYLTWNGYDWVNEDGSLFTKVKYVKRTVDITVLNNIFSYSNVVYKIVGDIDLGGGTLTIATGSTLDFQGGSFSNGTIVGNNTKIIAGL
3 WEY17523.1-Bc03  90.7%  47.1%        LDALTAKLDGTADRGYIVYITTFDSYLTWDGARWLNEDGSLFSKVKFIKATISIDTLNILFSY-NVTYKIVGNIDLDGGTLTIATGSILDFQGGSFSNGTITLSNTELKGNV

                         cov    pid 1081           .         1         .         .         .         .         :         .         .         .         .
1 WEU69857.1-Bc11 100.0% 100.0%        TLSGIWIADSLSPEHF-GITGIDATQDTTYIQKALDCCSTINIKTVKLQNKIYQINSSLLIQSNIKLEGAT------------SRVWN----------------NGSATI
2 WEU69745.1-Bc01  91.9%  44.1%        ILTGTWNVEESYPEWF-GAVGNGVTNDTIALTKCINYFTVTSLSA-----RTYITDQIDDIPSFRTIKGCGKKSIIQANPSMELTNNYLLRTKDGGSGIIFSDFVLLGGSNS
3 WEY17523.1-Bc03  90.7%  47.1%        TLAGSCSNDITKLSWFTDDLANDYSANLQSLVNVTKVQGTVDIDS-----LSFIVNTTVNITKALTIRGNG------------TRNYY---------------------

                         cov    pid 1201           .         .         .         .         :         .         .         .         .         3         .
1 WEU69857.1-Bc11 100.0% 100.0%        GITVE-STGVEINDLK----IIGNTTNTGISFTNRSY---YFSGNRIITTGLG----IGFDIQSSWTYIFNLCRIEGGTIGFKIQEGTSGTFNSCVAFSC----SEYGFYVT
2 WEU69745.1-Bc01  91.9%  44.1%        GILLR-STSNDVNDQWDTRNIIQNVEIKNCYAASI-YIGTYQRENKIVNCFISHTTNVGINCNGTDNMIIG-CTVAGSHQEGIIIN-GNNRIDSCKCFGCGASTTETDKYAL
3 WEY17523.1-Bc03  90.7%  47.1%        SIRNKITTANDI--------VILNITSTQVRVEGITFIGAYSTLDPTTGVRLGTQALVSFS-NASNCTLRN-CNFLYSHIGALFTNSGIANIEDNNFAGCNAGLRLMASPDS

                         cov    pid 1321           .         .         :         .         .         .         .         4         .         .         .
1 WEU69857.1-Bc11 100.0% 100.0%        GCGTDGCKY----------GFYFTDACRGVTLSSCGCENTQVG---GYMVKCGARA---------------------YVTITAFSVGTMADVDCDLFIFEADCRVSLIDL
2 WEU69745.1-Bc01  91.9%  44.1%        KCNVSNVELQQNNYG-----GCIIQGNSNYVQV---TCDNNGSG-TTTTVIGCNVVGSYNTVIVTSYNFSFHDDNYERYFVSTNWNTVGNNIIINGTPLANNKMATLSPWSI
3 WEY17523.1-Bc03  90.7%  47.1%        NTNSGNVTFSEDNAGTLNGVGLSINASSGNINVIGGKCEWNNKGMVIANSMGVAIIG----------------------VQFDWNKSG-NVIYNQT----------SYSL

                         cov    pid 1441           :         .         .         .         .         5         .         .         .         .         :
1 WEU69857.1-Bc11 100.0% 100.0%        YHPTGNTLNVASGAAVALINCYLTGTSV-----GLENCSVISPYGNTLKYSTTVDKINTKELVTSSLATNATYTVLSTAPLNS-----VY----LITAVTNGNSA-------
2 WEU69745.1-Bc01  91.9%  44.1%        WNGFNLTQARETPFNKYLVPNKYTTNGTGTFIQNDCGLAFRIASATAINADVLSYSFSIPNAI--MIDNNGCFSVKARFHKDSDVNSIIYPVIRVITTYTNSSGSSITRTDQ
3 WEY17523.1-Bc03  90.7%  47.1%        SHSITGCRFLGTPADQHIYIRYWSAANL-----NVCGCTFTKNASDVTAIDTSTGANEGPESIFKVVQKQEADEYRTKIHINFKGNT-IYRVVNLVNAVIQSTTPG---SGQ

                         cov    pid 1561           .         .         .         6         .         .         .         .         :         .         .
1 WEU69857.1-Bc11 100.0% 100.0%        ------------------------------------ARGLAIV------TIPTDYGMASVDNLVANANCQISIDATGNIIVTNTSTSAKAYRVSLLKLV------------
2 WEU69745.1-Bc01  91.9%  44.1%        NKDSLDVYALTSYHYLDDKPVSINSIIVEFVVMSKAIASGLAINAYFDDIRIGATTAGDKIAFLNEKGITSARPT------LALVSAGFKYYDTTLNKYIMSNGTAWTNLDG
3 WEY17523.1-Bc03  90.7%  47.1%        ------------------------------------ADGQTLG------KIGSCTIINNIDV--AKGTSQWRETAIVTNNLSTDDAGLTYFDTDLNKLVLWNGTAWTNVDG
```

MView 1.63, Copyright © 1997-2018 Nigel P. Brown

Fig. S4. Multiple sequence alignment of shared structural protein 2 from Fig 2C including 'K. tikkala' strain Bc01 protein, WEU69745.1, 'K. frurule' strain Bc03 WEY17523.1, and 'R. jaberico' strain Bc11 WEU69857.1 (reference sequence), showcasing the sequence identity with amino acids that were not shared in grey, and the rest in different color, based on the amino acid group.

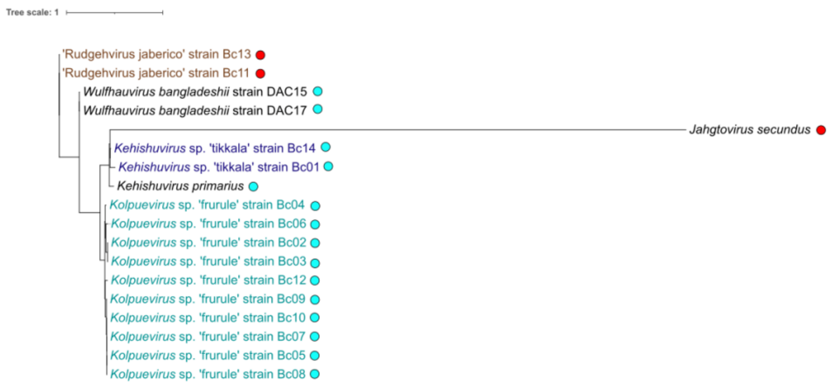

Fig. S5. Species tree inferred from orthogroups using OrthoFinder and rooted to 'Rudgehvirus jaberico' strain Bc13 using the STRIDE algorithm. The 14 *Crassvirales* isolates are color coded based on their species classification, 'Rudgehvirus jaberico' strains in brown, *Kehishuvirus* sp. 'tikkala' strains in dark blue, and *Kolpuevirus* sp. 'frurule' strains in light blue. The four *Crassvirales* genomes from other studies are color coded in black. Further, to denote the family level classification, we added red dots next to *Intestiviridae* family members, and cyan dots next to *Steigviridae* family members.
